# From chaos to kaleidoscope: Exploring factors in psychedelic self-treatment for mental health conditions

**DOI:** 10.1177/02698811241265762

**Published:** 2024-07-29

**Authors:** Claire Walker, Timothy Piatkowski, Jason Ferris, Emma Davies, Monica Barratt, Adam Winstock, Cheneal Puljević

**Affiliations:** 1 School of Psychology, The University of Queensland, Brisbane, QLD, Australia; 2 School of Applied Psychology and Center for Mental Health, Griffith University, Brisbane, QLD, Australia; 3 Center for Health Services Research, The University of Queensland, Brisbane, QLD, Australia; 4 Center for Psychological Research, Oxford Brookes University, Oxford, UK; 5 Social Equity Research Center and Digital Ethnography Research Center, RMIT University, Melbourne, VIC, Australia; 6 National Drug and Alcohol Research Center, UNSW Sydney, Sydney, NSW, Australia; 7 University College London, London, UK; 8 Global Drug Survey, London, UK; 9 School of Public Health, The University of Queensland, Brisbane, QLD, Australia

**Keywords:** Anxiety, depression, mental health, psychedelics, self-treatment

## Abstract

**Introduction:**

This study explores how individuals self-treat psychiatric conditions with psychedelics outside medical guidance bridging the gap in understanding unregulated therapeutic use.

**Aims:**

The primary objective was to extract specific factors underlying the effects of psychedelics, exploring their relationship with the need for medication, particularly for mental health conditions like depression and anxiety. Additionally, we aimed to understand how the likelihood of being prescribed pharmacological medication varies based on mental health diagnoses and demographic factors.

**Methods:**

This research utilised the Global Drug Survey 2020, an annual online survey focused on substance use patterns and demographics, incorporating modules addressing mental health and psychedelic use. The study employed Exploratory Factor Analysis to discern latent factors underlying the self-reported effects of psychedelics. Bivariable and multivariable logistic regressions were conducted to investigate the association between identified factors and the likelihood of current prescribed medication usage.

**Results:**

In all, 2552 respondents reported using psychedelics for self-treatment of mental health conditions. Three significant factors were identified: Improved Mental Health, Improved Self-Awareness and Neuro-Sensory Changes. The majority of the sample reported a history of depression (80%) or anxiety (65.6%), with a significant association observed between reported factors of psychedelics’ effects and current medication usage for mental health, especially notable in cases of depression or comorbid depression and anxiety.

**Conclusions:**

Perceived symptom improvement following psychedelic self-treatment may reduce the need for medically supervised pharmacological interventions. These findings highlight the potential of psychedelics to positively influence mental health and self-awareness, paving the way for further research into their therapeutic application.

## Introduction

Mental health conditions, posing substantial societal and personal challenges, often see moderate short-term benefits from traditional interventions like cognitive behavioural therapy (Cuijpers et al., 2013). Standard interventions like cognitive behavioural therapy and pharmacotherapy for depression offer short-term relief but often result in high relapse rates (Cuijpers et al., 2013; Vittengl et al., 2007). With variable success rates and widespread negative impacts of mental health conditions (Kamenov et al., 2017), investigating novel treatments like psychedelic compounds is crucial. Pharmacotherapies like selective serotonin reuptake inhibitors (SSRIs) alleviate depressive symptoms and negative cognitions but may also cause adverse reactions and withdrawal effects (Chouinard and Chouinard, 2015; Cipriani et al., 2018; Harmer and Cowen, 2013; Harmer et al., 2017; Henssler et al., 2019). Considering these limitations, the exploration of novel and effective treatment options is crucial (Kamenov et al., 2017). Psychedelic compounds offer promise in this regard, with an increasing global scientific interest in their therapeutic potential (Anderson et al., 2019; Basedow and Kuitunen-Paul, 2022; Cavarra et al., 2022; Garcia-Romeu and Richards, 2018; Rosenbaum et al., 2020). Examples of psychedelic substances explored in extant work include lysergic acid diethylamide (LSD), psilocybin (‘magic mushrooms’), MDMA (3,4-methylenedioxy-methamphetamine), N,N-dimethyltryptamine (DMT), ketamine, cacti such as mescaline, ibogaine and ayahuasca (Dobkin, 1968; Nichols, 2016; Studerus et al., 2010; Nyongo Ndoua and Vaghar, 2018).

Clinical trials suggest psychedelics offer promise for mental health and substance use disorders with minimal side effects compared to traditional medications (Andersen et al., 2021; Bogenschutz et al., 2022; Carhart-Harris et al., 2016; Davis et al., 2020; Mitchell et al., 2021). Research highlights their effectiveness in treating conditions like depression, anxiety, obsessive-compulsive disorder and substance use disorders, with long-lasting effects (Andersen et al., 2021; Bedi, 2018). MDMA trials show significant reductions in posttraumatic stress disorder symptoms (Smith et al., 2022), and reviews note positive effects on existential well-being, quality of life and mental health in terminal illness patients (Schimmers et al., 2022). Psychedelics also exhibit good tolerability and safety profiles in clinical settings (Andersen et al., 2021; Dos Santos et al., 2016), while non-clinical studies associate them with positive behaviour change and improved well-being (Garcia-Romeu and Richards, 2018; Jungaberle et al., 2018; Nayak et al., 2023; Teixeira et al., 2021). The emerging promise of psychedelics, despite regulatory, and scepticism barriers(Breeksema et al., 2022), suggests a potential breakthrough, but delays in translating evidence into practice may drive individuals towards self-exploration for alternative solutions.

### The current study

In the context of increasing awareness of the positive effects of psychedelics, and the illegal nature of psychedelic substances in most countries, there are growing reports of individuals self-treating mental health conditions with psychedelic substances (e.g. Hutten et al 2019; Kopra et al., 2022a, 2022b; Lawn et al 2017; Nayak et al., 2023; Pestana et al., 2021). For example, three studies using data from the Global Drug Survey (GDS), the world’s largest annual online survey of drug use, have explored survey respondents’ experiences of using psychedelic substances to self-treat mental conditions. A study by Lawn et al. (2017), using data from the 2015 to 2016 GDS, found that ayahuasca users (*n* = 527) reported better well-being than comparison groups (*n* = 78,236) and less problematic drinking than classic psychedelic users (*n* = 18,138). Two studies by Kopra et al (2022a, 2022b) aimed to investigate the incidence and nature of seeking emergency medical treatment (EMT) among GDS respondents who reported using psilocybin (Kopra et al., 2022a) or LSD (Kopra et al., 2022b). Both studies found very low numbers of respondents reporting seeking EMT after using psilocybin (*n* = 19; 0.2%) or LSD (*n* = 102; 1.0%), with these respondents most commonly reporting psychological symptoms (e.g. anxiety) as the reason for seeking EMT (Kopra et al., 2022a, 2022b). A recent study by Nayak and colleagues (2023), surveying naturalistic psilocybin use, explored participants’ self-reported experiences at three timepoints before and three timepoints after psilocybin use, with participants typically reporting lasting improvements in mental health symptoms and general well-being. Other studies exploring individuals’ experiences of self-treating mental conditions or other symptoms are largely limited to people who microdose psychedelics (e.g. Hutten et al., 2019; Lea et al., 2020), with these participants self-reporting predominantly positive outcomes.

Using data from the GDS, the present study seeks to contribute to this body of research exploring individuals’ experiences of self-treating mental conditions using psychedelic substances by identifying the factors that underly the self-reported negative or positive effects of psychedelic substances that respondents describe within the past 12 months. Inspired by recent research by Nayak et al. (2023), this study has two secondary aims; first to explore the associations between the identified factors and respondents’ reported use of prescribed psychiatric medication to manage their mental health conditions (specifically relating to anxiety or depression); and second, to investigate variations among respondents based on their specific mental health diagnosis of depression, anxiety or both.

## Methods

### Design and sample

This study uses data from the GDS, an annual anonymous cross-sectional online survey of drug use. To participate in the GDS, respondents must be at least 16 years old and have used at least one drug (including alcohol and tobacco) in the past 12 months. Respondents are recruited through promotion of the survey by worldwide media and organisational partners. Details about the GDS’s methodology, including survey design, recruitment and representativeness have been previously described (Barratt et al., 2017; Winstock et al., 2022). For inclusion in these analyses, there was a specific section that contained information around the use of psychedelics for self-treatment of a psychiatric condition. Only respondents who responded to this specific section and provided a valid response to each of the 17 items used in the exploratory factor analysis were included for analysis resulting in analysis based on 2552 respondents. Tables 1 and 2 report any item missingness across the socio-demographics and characteristics relating to the use of psychedelic substance. For the logistic regression models, the outcome variable ‘are you currently prescribe medication to treat your mental illness’ was not answered by 510 people as they had indicated not ever being ‘prescribed medication to treat the symptoms of mental illness’; a further three respondents were excluded from the model as they did not provide a valid answer to the outcome question. This resulted in the logistic regression analysis being based on 2000 respondents.

**Table 1.** Respondents’ socio-demographic characteristics.

Characteristic	(*n* = 2552), number (%) [missing]
Age**—**years (mean ± SD)	26.3 (8.8)
Gender	
Male	1543 (60.5)
Female	880 (34.5)
Non-binary	96 (3.7)
Different identity	33 (1.3)
Ethnicity	
Caucasian	2078 (81.9)
Hispanic/Latino	146 (5.8)
Mixed ethnicity	184 (7.3)
South-East Asian (including Chinese, Vietnamese, Japanese, Thai)	28 (1.1)
Asian (including Pakistani, Indian, Bangladeshi)	16 (0.6)
African American	8 (0.3)
African/Caribbean	8 (0.3)
Aboriginal, Torres Strait Islander or Māori	8 (0.3)
Indigenous American	10 (0.4)
Other	50 (2.0)
	[16]
Country of residence	
United States of America	645 (25.3)
Germany	356 (13.9)
Australia	274 (10.7)
England	176 (7.0)
Finland	223 (8.7)
Brazil	148 (5.8)
Canada	111 (4.3)
Denmark	53 (2.1)
Russian Federation	36 (1.4)
Mexico	32 (1.3)
Netherlands	46 (1.8)
Austria	43 (1.7)
Other^‡^	409 (16.0)
Education^†^	
Tertiary qualification	1,415 (55.8)
Higher secondary school	633 (24.9)
Less than higher secondary school	426 (16.8)
No formal schooling	17 (0.7)
Don’t know	46 (1.8)
	[15]
Ever diagnosed with mental health condition*	
Depression	2040 (80.0)
Anxiety	1675(65.6)
ADHD	669 (26.2)
PTSD	526 (20.6)
Bipolar disorder	332 (13.0)
Psychosis	160 (6.3)
Other	504 (19.8)
Comorbid mental health condition	
Depression and anxiety	1471 (57.6)
Ever been prescribed medications for mental health condition	
Yes	2003 (78.5)
	[39]
Currently taking prescribed medications for a mental health condition	(*n* = 2003)
Yes	954 (47.7)
	[3]

* Multiple responses were allowed.

† Tertiary qualifications include the following: technical or trade certificate, college certificate/diploma, undergraduate or postgraduate degree; less than higher secondary school includes: primary school and lower secondary school.

‡ Consists of countries where responses account for <2% of the total sample.

**Table 2.** Characteristics surrounding the use of psychedelic substances.

Characteristic	(*n* = 2552), number (%) [missing]
Substance/s used over the previous 12 months to manage a psychiatric condition*	
LSD	1405 (55.1)
Psilocybin (‘magic mushrooms’)	1165 (45.7)
MDMA	1045 (41.0)
Ketamine	630 (24.7)
DMT	337 (13.2)
Ayahuasca	150 (5.9)
Peyote	26 (1.0)
Ibogaine	19 (0.7)
Also use a psychedelic substance to address a specific worry/concern in your life (e.g. relationship issue, bereavement, addiction, trauma)	
No, never	297 (11.7)
Yes, in the last 12 months	2126 (83.8)
Yes, not in the last 12 months	114 (4.5) [15]
Main psychiatric or other condition attempting to treat when using these substances	
Depression	1157 (46.1)
Anxiety	444 (17.7)
PTSD	159 (6.4)
Relationship problem	151 (6.0)
Trauma	134 (5.4)
Alcohol or other substance use disorder	85 (3.4)
Bipolar disorder	69 (2.7)
Bereavement	45 (1.8)
Distress associated with a medical disorder	32 (1.3)
Anorexia/bulimia	20 (0.8)
Psychosis	18 (0.7)
Obsessive-compulsive disorder	16 (0.6)
Chronic pain	14 (0.6)
Over-eating	11 (0.4)
Cancer-related mental health distress	8 (0.3)
Other	146 (5.8) [43]
**Last 12 months: number of days used psychedelic substance for treatment of the condition (median** **±IQR)**	**3 (2–10) [26]**
**Last 12 months: Used the substance on other occasions for purely recreational purposes**	1766 (69.6) [16]
**Last 12 months: number of days taken the substance for recreational purposes (** **median ±** **IQR)**	5 (2–12) [30]
**Description of the maximal short-term effects of the dose usually taken for therapeutic purposes**	
Intense psychedelic experience	1068 (42.3)
Moderate psychedelic experience	981 (38.8)
Mild psychedelic experience	371 (14.7)
No psychedelic experience, but other effects	87 (3.5)
No experience or effects at all	19 (0.7) [26]

IQR: interquartile range.

* Percentages can exceed 100 as the response option was multiple choice.

GDS2020 ran from November 2019 to January 2020 and was translated into 19 languages (English, Albanian, Azerbaijani, Brazil, Czech, Danish, Dutch, Finnish, French, German, Hungarian, Italian, Lithuanian, Portuguese, Romanian, Serbian, Slovak, Spanish and Turkish). This study was pre-registered on the Open Science Framework (https://osf.io/8htwq/) and received ethical approval from The University of Queensland (2017001452), University College London Research Ethics Committee (No: 141/02) and The University of New South Wales (HREC HC17769).

### Measures

Each GDS includes core modules that collect data on socio-demographic characteristics (self-reported age, gender, country of residence, ethnicity, education level), drug use history (drug use in past 30 days, 12 months or ever) and mental health (previous diagnosis of mental health conditions (depression, anxiety, ADHD, PTSD, bipolar disorder, psychosis, other; medication(s) prescribed and used for mental health disorders) and several specialist modules collecting data on topical issues. GDS2020 included a specialist module on psychedelic use for self-treatment of a psychiatric (specific worry or concern) or other condition; questions covered psychedelic substances used in the past 12 months to manage a psychiatric condition (response options: LSD, psilocybin, MDMA, ketamine, DMT, ayahuasca, peyote or ibogaine); previous use of a psychedelic substance to address a specific worry or concern (never; yes in the past 12 months; yes but not in the past 12 months); the main psychiatric condition or symptom respondents were attempting to treat with psychedelic substances (depression; anxiety; PTSD; relationship problem, trauma, substance use disorder; bipolar disorder; bereavement; distress associated with a medical disorder; anorexia/bulimia; psychosis; obsessive compulsive disorder; chronic pain; over-eating; cancer-related mental health distress; other); number of days the psychedelic substance was used to treat the condition; use of a psychedelic substance for purely recreational purposes (yes/no); number of days the psychedelic substance was used purely for recreational purposes); and description of the maximal short-term effects of the dose usually taken for therapeutic purposes (intense psychedelic experience; moderate psychedelic experience; mild psychedelic experience; no psychedelic experience but experienced other effects; no experience or effects at all). The main item of interest in this module was the following question: ‘Which of the following effects have you noticed as a result of your use of this psychedelic substance over the last 12 months on a scale of −3 to +3? (e.g. −3 = strong negative consequences; 0 = no change; +3 = strongly positive; N/A = not applicable)?’ Response options are listed in Appendix A. All questions were developed by the authors and other experts with experience in research on psychedelic substances.

### Data analysis

All data were initially analysed using descriptive statistics. We then used exploratory factor analysis (EFA) as a variable reduction technique to understand the common factors that explain the order and structure of respondents’ perceived effects when using psychedelics for self-treatment of mental health conditions or other symptoms. As a Likert scale was used to measure respondents’ perceived effects of psychedelics, with response options ranging from −3 (strong negative consequences) to +3 (strong positive consequence), we recoded response options on a scale of 1–7, reflecting the range of negative to positive consequences, to aid interpretation of the EFA. Cronbach’s alpha was used to calculate internal consistency reliability. For the EFA stopping rule, Kaiser’s (1958) Criterion and Scree Plot were used given that the analysis was exploratory with nil firm a priori ideas. Principal axis factoring was employed to explore the structure of a latent variable set, considering potential correlations among factors, and an oblique rotation method (Promax) was chosen due to the research focus area (Grieder and Steiner, 2022). Given the number of respondents, criteria for determining salient factor loadings were set at ⩾0.40 (Stevens, 2012). Salient item loadings on more than one factor, or complex item loadings on all factors which included a low salient item factor loading, were rejected to honour a simple factor structure (Thurstone, 1931). The final model (presented in Table 3) presents the results of the EFA with these specific items removed from the analysis; this final model is the model used to create the factors for additional analysis.

**Table 3.** Pattern coefficients for the effects noticed resulting from the use of psychedelics for self-treatment of a psychiatric condition or emotional distress.

Initial model	Loadings					
	Initial model			Final model		
Item	Factor 1^a^	Factor 2^b^	Factor 3^c^	Factor 1^a^	Factor 2^c^	Factor 3^b^
Changes in mood or reduced depression	**0.97**	−0.15	−0.02	**0.98**	−0.03	−0.13
Change in overall symptoms of your psychiatric condition	**0.89**	−0.09	−0.00	**0.89**	−0.02	−0.06
Changes in ability to control negative thoughts/persistent worrying	**0.73**	0.02	0.05	**0.67**	0.05	0.08
Changes in productivity, motivation or confidence	**0.64**	0.12	0.04	**0.59**	0.09	0.12
Changes in feelings of frustration/anger	**0.45**	0.20	0.12	0.39	0.10	0.28
^†^Changes in energy, alertness and/or focus	**0.44**	**0.42**	−0.10	—	—	—
Changes in anxiety, including social anxiety	**0.40**	0.35	0.01	0.35	−0.02	**0.44**
^‡^Changes in my tolerance towards others	0.35	0.15	0.24	—	—	—
Changes in my understanding of why I feel the way I do	0.03	−0.15	**0.90**	0.01	**0.93**	−0.14
Changes in my understanding of my condition or how I relate to it	0.02	−0.11	**0.87**	0.00	**0.89**	−0.09
Changes in self-identity	−0.02	0.18	**0.57**	0.00	**0.54**	0.18
Changes in life priorities	−0.04	0.29	**0.53**	0.04	**0.50**	0.24
Changes in empathy, sociability and communication skills	0.13	0.22	**0.41**	0.07	0.38	0.30
Changes in concentration/memory	−0.02	**0.78**	−0.06	−0.05	−0.05	**0.79**
Changes in sleep	0.02	**0.59**	−0.05	0.06	−0.07	**0.55**
Changes in sight, smell or hearing	−0.06	**0.45**	0.08	−0.06	0.08	**0.46**
^‡^Changes in my use of alcohol/other drugs	−0.00	0.35	0.20	—	—	—
Explained variance	43.38%	5.13%	2.94%	44.31%	5.97%	3.36%

† Loading on more than 1 factor.

‡ Factor loading <0.4 on any factor.

Note. a = “Improved mental health”; b = “Improved self-awareness” c = “Neuro-sensory changes”. Bolded numbers indicate which results loaded onto a factor (0.40+)

Following the identification of the latent factors using EFA, novel variables reflecting these factors were calculated using the mean sum of scores method to retain the scale metric and aid factor interpretation. This approach is appropriate as items loading across more than one factor are removed (DiStefano et al., 2009; Loewen et al., 2015). The EFA is based on a sample of respondents who met the following three criteria: reported using at least one of the eight psychedelic drugs (LSD, psilocybin, ketamine, MDMA, peyote cacti, DMT, ayahuasca or ibogaine), reported a diagnosis of a mental health condition and reported using a psychedelic drug to manage a diagnosed psychiatric condition, all in the past 12 months. When respondents reported ‘not applicable’ or did not provide an answer to the item included in the EFA, data were treated as missing values. As this study is exploratory, missing data were addressed through listwise deletion. The final sample size for the EFA was 2552. This sample size exceeds the minimum sample of 300 respondents recommended for EFA by Tabachnick and Fidell (2007), as well as the threshold of 1000 respondents considered as ‘excellent’ for an EFA (Comrey and Lee, 2013). Furthermore, with 2552 respondents we had a case to variable ratio of 1:150, exceeding the adequate ratio of 1:10 or higher recommended by Costello and Osborne (2005).

Regarding the secondary aim of the study, the analysis was restricted to the 2003 respondents (see Table 1) who reported a valid response (yes/no) to currently being prescribed medication to treat their mental health diagnosis. Prior to modelling the three bivariable logistic regression models, LOWESS smoother curves were explored to determine if the factors required modelling in linear or non-linear forms (see Appendix B). There was a clear curvilinear association between Improved Mental Health and currently prescribed medication as such when modelling the bivariable association between currently prescribed medication and Improved Mental Health this was modelled as a quadratic term to account for the ‘inverted-U’ shape (see in Appendix B). While the LOWESS smoother curve for the improved self-awareness factor is not as clearly linear (or monotonic) as the Neuro-Sensory factor both were modelled as linear terms. The data indicate insignificance of the quadratic term for improved self-awareness, and therefore the linear term was used.

Therefore, to address the secondary aims of the study, three bivariable logistic regression analyses were undertaken to assess the association between each of the identified factors and the dichotomous outcome variable of currently prescribed medication (yes/no) to treat their mental health condition (specifically anxiety or depression). Following this, three multivariable logistic regression models were undertaken to explore the differences in the association between the factors of the specific mental health conditions of anxiety, depression or both. All logistic regression models were conducted using complete case analyses. We used Stata V18 for all analyses, with Stata’s margins and margins plot commands utilised to visualise the results. In line with common practice in public health and behavioural research, we employed a conventional significance level of α < 0.05 for our analyses. Exact *p*-values are presented where *p* > 0.001.

## Results

Table 1 shows the socio-demographic characteristics of the 2552 respondents included in this study’s analyses. Respondents were aged between 16 and 72 years old, with a mean age of 26.3 years. The majority of respondents described themselves as male (60.5%) and Caucasian (82%), and as living in Western countries; respondents most commonly reported living in the USA (25.3%), Germany (13.9%) or Australia (10.7%). A majority of the sample reported previous diagnoses of a mental health condition, including depression (80.0%) and anxiety (65.6%), with 57.6% of the sample reporting a diagnosis of both depression and anxiety. Almost 80% of the sample (78.5%) reported being prescribed medication for their mental health condition; however, less than half (47.7%) were currently taking prescribed medication.

Table 2 shows respondents’ use of psychedelic substances to manage a diagnosed psychiatric condition in the last 12 months. Respondents most commonly described using LSD (55.1%), followed by psilocybin (45.7%) and MDMA (41.0%). The majority of the sample (83.8%) also reported using psychedelics to address a specific worry or concern in their life in the past 12 months. Respondents reported that depression (46.1%) or anxiety (17.7%) were the main diagnosed psychiatric condition or symptom that they were attempting to treat when using psychedelics. In the last 12 months, the median number of days on which respondents had used a psychedelic substance for treatment of a condition was three (ranging between 1 and 365). Most respondents (69.6%) indicated they had also used the same psychedelic drug in the last 12 months for recreational purposes; the median number of days, in the past 12 months, that respondents had used the same psychedelic substance for recreational purposes was five (again, ranging between 1 and 365). Approximately four out of five respondents (81.1%) reported experiencing an intense or moderate short-term effect from the psychedelic substance, with less than 5% reporting no psychedelic experience.

### Factor structure

The EFA demonstrated high internal consistency reliability (α = 0.91). Three factors (see Table 3; Initial Model), all with eigenvalues > 1, explained 51.5% of the variance in the 17 questionnaire items. Including items with loadings ⩾0.04, factor 1 (7 items) had a Cronbach’s alpha of .90, Factor 2 (4 items) had .65 and Factor 3 (5 items) had 0.86. The correlations between each set of Factors were as follows: 1 and 2 *r* = 0.76, Factors 1 and 3 *r* = 0.68 and Factors 2 and 3 *r* = 0.66. The alpha values suggest moderate to good reliability and internal consistency of associations between the grouped items within each factor. While the factors are quite distinct, given the reported correlation values, the factors do share a substantial amount of variation. Undertaking sensitivity analysis for items that did not load well onto a factor confirmed similar results. Items removed for sensitivity analysis included ‘changes in energy, alertness and/or focus’ due to loadings of ⩾0.40 on more than one factor and both ‘changes in my tolerance toward others’ and ‘changes in my use of alcohol and other drugs,’ due to poor factor loading. The final model in Table 3 presents the revised EFA; the three factors now account for 53.6% of the variance in the 14 items. Factor 1 (4 items) had a Cronbach’s alpha of 0.88, Factor 2 (4 items) had 0.84 and Factor 3 (4 items) had 0.70. The correlations between each set of Factors were as follows: 1 and 2 *r* = 0.67, Factors 1 and 3 *r* = 0.74 and Factors 2 and 3 *r* = 0.67.

Based on the final model, the three factors were named *Improved Mental Health, Improved Self-Awareness* and *Neuro-Sensory Changes.* The factor *Improved Mental Health* incorporates significant shifts in mood, mental health or psychiatric disturbance; *Improved Self-Awareness* includes transformations in self-perception and self-reflection; and *Neuro-Sensory Changes* encompasses modifications in cognitive and sensory processes (but also anxiety which reasonably would be associated with concentration and changes in sleep and sensory experiences). Table 4 presents summary statistics for each of the three factors. On the seven-point scale, the factors of *Improved Mental Health* and *Improved Self-Awareness* had mean scores of 5.56 and 5.52, respectively, reflecting respondents’ overall positive experience of using psychedelic substances for these perceived effects, while the score of 4.61 for *Neuro-Sensory Changes* indicates no or little perceived positive effect for these changes.

**Table 4.** Means and standard deviations of the three detected factors (final model).

Factor	*M*	SD	Median	p25–p75
Improved mental health (4 items)	5.56	1.12	5.75	5.00–6.50
Improved self-awareness (4 items)	5.52	1.03	5.50	4.75–6.25
Neuro-sensory changes (4 items)	4.61	0.91	4.50	4.00–5.25

Range score 1–7 (1 = strong negative effects, 4 = no change, 7 = strong positive effects).

### Factors and current medication usage for mental health conditions

**Figure 1.**
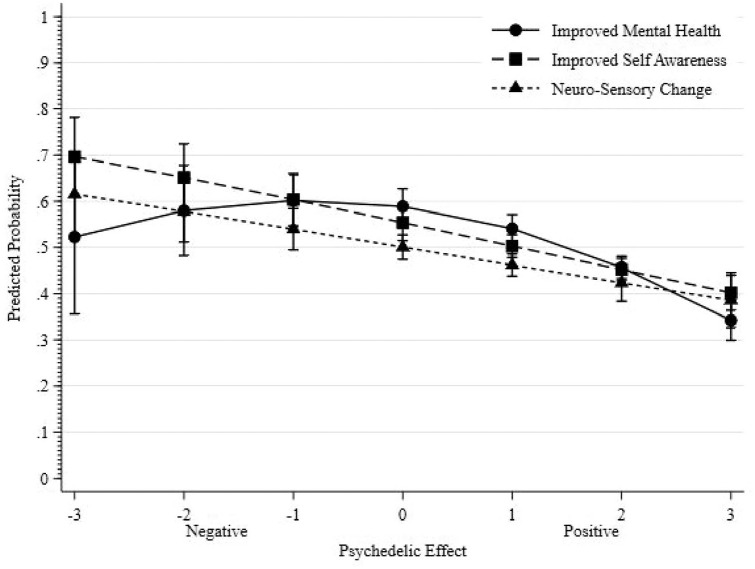
Predicted probability of reporting current medication prescription for mental health diagnosis modelled separately for each of the three factor scores. *Across the three factors, due to a low number of observations and model uncertainty with respect to −3 the confidence bound of the predicted probabilities extends beyond the logical range 0–1; this has been truncated to 0 and 1, respectively.

As shown in Table 5, all three factors were significantly associated with the odds of respondents having a current medication prescription to treat their mental health condition. Higher reported factor scores, indicating a more positive impact of psychedelics, were associated with a lower likelihood of respondents having a current medication prescription for their mental health concerns. This association was evident for both Improved Self-Awareness and Neuro-Sensory Changes (as depicted in Figure 1). However, there was a notable curvilinear relationship between the Improved Mental Health factor and the odds of reporting a current medication prescription. As scores indicating the negative effects of psychedelics shift towards neutral or weakly negative effects, the likelihood of respondents reporting a current medication prescription for mental health conditions rises. Conversely, as scores move from neutral ‘no change’ effects towards strongly positive effects, the odds of having a current medication prescription for mental health conditions decrease.

**Table 5.** Bivariable logistic regression (*n* = 2000*): odds ratio for current medication prescription for each new factor: improved mental health, improved self-awareness and neuro-sensory changes.

Factor	OR	95% CI		*p*-value
		Lower	Upper	
Improved mental health	1.561	1.029	2.369	0.036
Improved mental health^2^	0.931	0.892	0.971	0.001
Improved self-awareness	0.815	0.747	0.890	<0.001
Neuro-sensory changes	0.856	0.775	0.944	0.002

* Listwise deletion resulted in three people being excluded as they did not provide a valid answer to the outcome question 2the quadratic form of the factor modelled.

**Figure 2.**
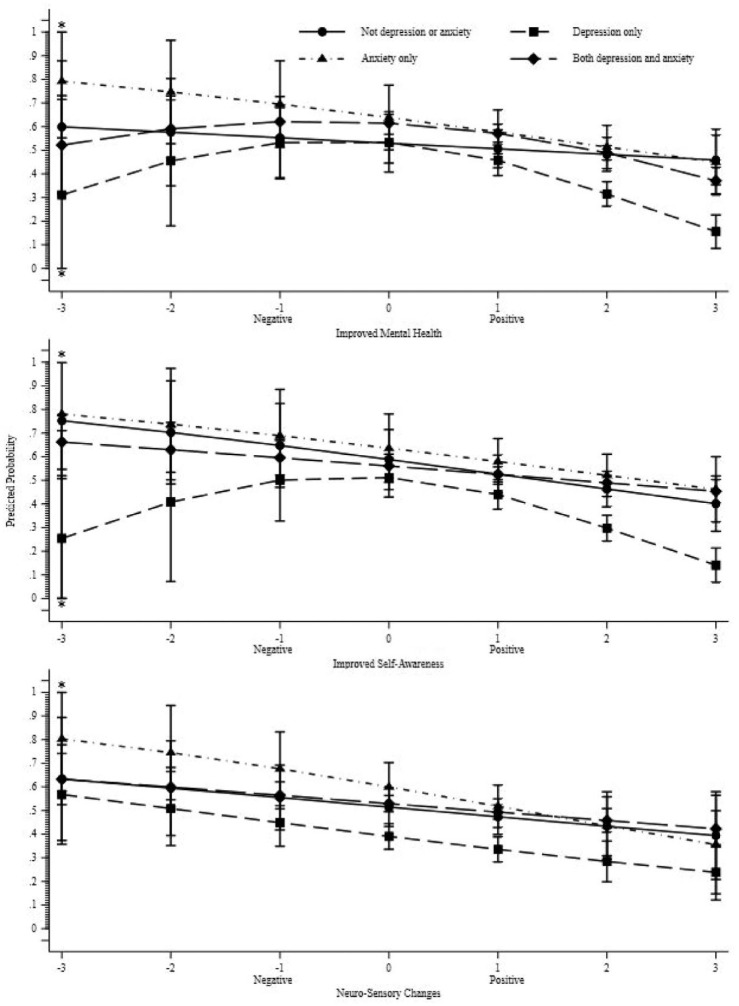
Separated by each of the three factors: Predicted probability of reporting current medication prescription for respondents diagnosed with depression only, anxiety only, both depression and anxiety and neither depression nor anxiety. *Across the three factors, due to a low number of observations and model uncertainty with respect to −3 the confidence bound of the predicted probabilities extends beyond the logical range 0–1; this has been truncated to 0 and 1, respectively.

Table 6 and Figure 2 explore the bivariable associations between each of the three factors and the odds of respondents having a current medication prescription to treat their mental health conditions, for respondents who reported a mental health concern of depression only (*n* = 421), anxiety only (*n* = 136), comorbidity of depression and anxiety (*n* = 1241) and a mental health concern that was neither depression nor anxiety (*n* = 202). Across all three factors, there was no evident link between each factor and the likelihood of having a current medication prescription for mental health conditions in respondents with other mental health conditions besides anxiety or depression. For respondents who reported a mental health condition that was neither depression nor anxiety the more positive the effect of psychedelics, the more likely that respondents reported *not* having a current medication prescription for their mental health concern (see the long-dashed line in the centre figure of Figure 2). The absence of a substantial link between each of the three factors and the disclosure of current medication prescriptions for mental health treatment may be influenced by the insufficient number of individuals who reported having neither depression nor anxiety or only anxiety.

**Table 6.** Stratified bivariable logistic regression for each factor: odds ratio for current medication prescription for respondents diagnosed with depression only, anxiety only, both depression and anxiety and neither depression nor anxiety (*N* = 2000).

Factor	Not depression or anxiety (*n* = 202)	Depression only (*n* = 421)	Anxiety only (*n* = 136)	Both depression and anxiety (*n* = 1241)
	OR (CI); *p*-value	LL	UL	*p*-value
Improved mental health	0.910 (0.715–1.159); 0.444	2.928 (0.906–9.464); 0.073^a^	0.773 (0.567–1.056); 0.106	1.666 (0.986–2.814); 0.057^b^
Improved mental health^2^	NA	0.858 (0.763–0.965); 0.011^a^	NA	0.926 (0.878–0.977); 0.005^b^
Improved self-awareness	0.777 (0.587–1.030); 0.079	3.324 (0.758–14.585); 0.111^c^	0.788 (0.572–1.088); 0.148	0.867 (0.776–0.969); 0.012
Improved self-awareness^2^	NA	0.848 (0.733–0.980); 0.026^c^	NA	NA
Neuro-sensory changes	0.850 (0.628–1.150); 0.291	0.787 (0.618–1.002); 0.052	0.716 (0.496–1.034); 0.075	0.867 (0.766–0.982); 0.024
Neuro-sensory changes^2^	NA	NA	NA	NA

NA: not appropriate as the quadratic term was non-significant and including it would over-specify the model. 2the quadratic form of the factor modelled

a Adjusted Wald test for the fitted model: $X_{\left(1\right)}^{2}=6.48;\;(p=0.011)$ .

b Adjusted Wald test for the fitted model: $X_{\left(1\right)}^{2}=7.99\;(p=0.005)$ .

c Adjusted Wald test for the fitted model: $X_{\left(1\right)}^{2}=4.97\;(p=0.026)$ .

There were significant associations between each of the three factors and reporting a current medication prescription for those with depression only or comorbidity of depression and anxiety (see Table 6). First, for respondents who reported depression only, there was a significant curvilinear relationship between Improved Mental Health and Improved Self-Awareness factors (which can be observed in Figure 2 by the short-dashed line). While this curvilinear association was not observed for the Neuro-Sensory Changes factor, the linear association between Neuro-Sensory Changes and current medication prescription was significant for this group. For respondents who reported comorbidity with both depression and anxiety (the largest group, *n* = 1241), a curvilinear relationship was only observed between the Improved Mental Health factor and reporting current medication prescription. The association between the remaining factors (Improved Self-Awareness and Neuro-Sensory Changes) and the outcome was significant in a linear form. Higher factor scores correlated with a greater likelihood of not having a current mental health medication.

## Discussion

This study aimed to identify underlying factors associated with the effects reported by individuals using psychedelics for self-treatment of psychiatric conditions. An EFA was conducted, and the final model revealed three key factors: Improved Mental Health, Improved Self-Awareness and Neuro-Sensory Changes, which accounted for 51.5% of the variance. The first factor primarily represented mood shifts and reductions in depression, suggesting its relevance to mood disorders. The second factor, Improved Self-Awareness, had a limited influence on the reported effects, aligning with cognitive-intellectual experiences. The third factor, Neuro-Sensory Changes, encompassed changes in behaviour, lifestyle and social anxiety, and sensory-aesthetic experiences. These factors provide a framework for understanding the multifaceted effects of psychedelics on individuals’ psychological and perceptual experiences.

These findings align with Strassman et al. (1994) in detecting two clusters of mental health symptom changes: affect and cognition. Furthermore, the study expands on the concept of emotional breakthroughs mediated by psychedelics, as described by Roseman et al. (2019), and highlights the element of Improved Mental Health in the use of psychedelics for self-treatment of mental illness. Additionally, the study identifies a factor of Improved Self-Awareness, which overlaps with previous research on ego dissolution and realignment of life priorities associated with psychedelic use (Griffiths et al., 2006; Hartogsohn, 2018; Lebedev et al., 2016). The Neuro-Sensory Changes factor in the current study differs from Strassman et al.’s (1994) Cognition factor, indicating the need for further investigation. Future research should explore the specific sensory and behavioural changes experienced by individuals using psychedelics for self-treatment of psychiatric conditions (Barbanoj et al., 2008; Froese et al., 2018; Lawn et al., 2017; Rolland et al., 2014).

As a secondary aim, this investigation delved into two aspects. First, we aimed to probe the link between identified factors and the likelihood of participants using prescribed medication for their mental health conditions. Additionally, it aimed to analyse variations in this likelihood based on respondents’ specific mental health diagnoses. The study demonstrated that higher factor scores, signifying a more positive impact of psychedelics, were linked to a reduced likelihood of having a current medication prescription for mental health conditions. In this way, these data also add weight to Nayak et al.’s (2023) work which showed that most participants in their study (>90%) viewed their naturalistic psilocybin use positively, and more than 80% attributed desirable changes in well-being and life satisfaction to their experience. However, a curvilinear relationship was observed between Improved Mental Health and medication likelihood, suggesting a nuanced association with the intensity of psychedelic effects. For individuals with depression or comorbid depression and anxiety, significant linear or curvilinear relationships were found between the factors and reporting current medication usage, highlighting potential variations based on specific mental health conditions. These findings substantiate previous research which has observed diminished acute subjective effects of psilocybin (Nayak et al., 2023), as well as LSD (Bonson et al., 1996), specifically in terms of challenging experiences, among individuals concurrently using drugs for depression. Conversely, in cases of neither depression nor anxiety, positive psychedelic effects were associated with a lower likelihood of having a current medication prescription for mental health conditions. This finding is consistent with Dos Santos et al.’s (2016) systematic review of clinical trials, which demonstrated the antidepressant and anxiolytic effects of substances like ayahuasca, LSD, and psilocybin, particularly in individuals who had limited success with traditional treatments. These findings may also share some semblance with longitudinal evidence involving patients with life-threatening cancer who underwent psilocybin-assisted psychotherapy which revealed that approximately two-thirds of patients experienced clinically significant reductions in depressive and anxiety symptoms (Agin-Liebes et al., 2020). The results underscore the complex interplay between psychedelic effects and mental health treatment, suggesting potential implications for medication use and treatment strategies.

The present study’s findings extend the currently limited evidence base and call for research on people self-medicating with these substances. Improvements in mental well-being and increased self-awareness resulting from psychedelic use suggest potential benefits for managing mental health conditions. Individuals experiencing symptom improvements were less likely to require pharmacological medication for depression or anxiety, indicating psychedelics as a potential alternative for those unresponsive to traditional treatments. Therefore, continued research on psychedelics in the context of mental health conditions is warranted. For instance, while the GDS offers a comprehensive understanding of contemporary psychedelic use patterns, the National Survey on Drug Use and Health (NSDUH) provides valuable population-based data on lifetime psychedelic use and associated clinical outcomes (Jahn et al., 2021; Sexton et al., 2019). While novel psychedelic use was not linked to psychological distress (Sexton et al., 2019) compared to classic psychedelics (Hendricks et al., 2015), it was associated with an increased likelihood of past-year suicidal thinking and planning relative to classic psychedelic use, suggesting potential differences in effects between these two categories of psychedelics (Sexton et al., 2020). Comparing findings from GDS and NSDUH surveys could elucidate similarities and differences in substance use behaviours and risk-taking tendencies among psychedelic users.

### Limitations

This study has several limitations that should be acknowledged. First, the online nature of the study introduced heterogeneity in terms of the types of psychedelics used, the psychiatric conditions being treated, and the frequency and purpose of use. Respondents were required to select one specific psychedelic substance for their responses, which may have limited the generalisability of their experiences, as individuals might have had varying encounters with different psychedelics. While this increased external validity, it compromised internal validity due to the broad differences among respondents and substances. We also acknowledge a lack of inquiry regarding the dosage of the psychedelic substance used, which could potentially impact the comprehensiveness of the findings. In addressing another potential limitation, it is important to acknowledge that the survey included questions about perceived effects resulting from psychedelic substance use over the last 12 months, even though these effects were based on expert opinion rather than established scales. Furthermore, the naturalistic design introduced confounds as respondents reported using other therapeutic interventions and medications, making it challenging to isolate the specific effects of psychedelics. Additionally, missing data were present for many variables, and the sample consisted primarily of internet-connected respondents from Western cultures, limiting generalisability. However, the study provides important insights into a cultural shift in mental health treatment driven by the community, despite legal restrictions on psychedelic use in many countries (Barnett et al., 2018; Cavarra et al., 2022). Furthermore, we acknowledge some of the more cautious discussions regarding psychedelics within the scholarly community. For example, Breeksema et al. (2022) have emphasised the need for a more thorough examination of adverse events in clinical treatments involving serotonergic psychedelics and MDMA. Additionally, other research has also underlined a call for a more comprehensive assessment of harms in psychedelic-assisted therapy, underscoring the need for a more nuanced understanding of the potential risks associated with these interventions (Devenot et al., 2022; McNamee et al., 2023). Lastly, we did not collect data on the specific types of medication used by participants. While the study focused on medication as a key indicator of pharmacotherapy’s influence on biochemistry and cognition, this approach may have overlooked the potential impact of other interventions such as psychotherapy or counselling, as well as different care settings like outpatient, inpatient or intensive outpatient programs. Additionally, future research employing experimental designs could incorporate controls to investigate whether respondents who did not receive medication or other treatments were treatment-seeking individuals lacking access.

## Conclusions

This study aimed to identify the underlying factors associated with the effects experienced by individuals using psychedelics for self-treatment of psychiatric conditions or psychological distress. Utilising data from the GDS2020, three key factors emerged: Improved Mental Health, Improved Self-Awareness and Neuro-Sensory Changes. The investigation into the relationship between these factors and the likelihood of requiring pharmacological medication for depression or anxiety revealed that changes in Improved Mental Health were associated with a decreased need for medication in both conditions. Considering the high prevalence and significant impact of depression and anxiety on individuals’ well-being, further research exploring the therapeutic potential of psychedelics in mental health treatment is warranted. As part of the broader effort to address the global burden of these conditions, alternative treatment modalities should be carefully considered. The diverse range of effects observed in individuals utilising psychedelics for self-treatment presents a unique opportunity to contribute to the field of mental health intervention.

## Figures and Tables

**Appendix B.**
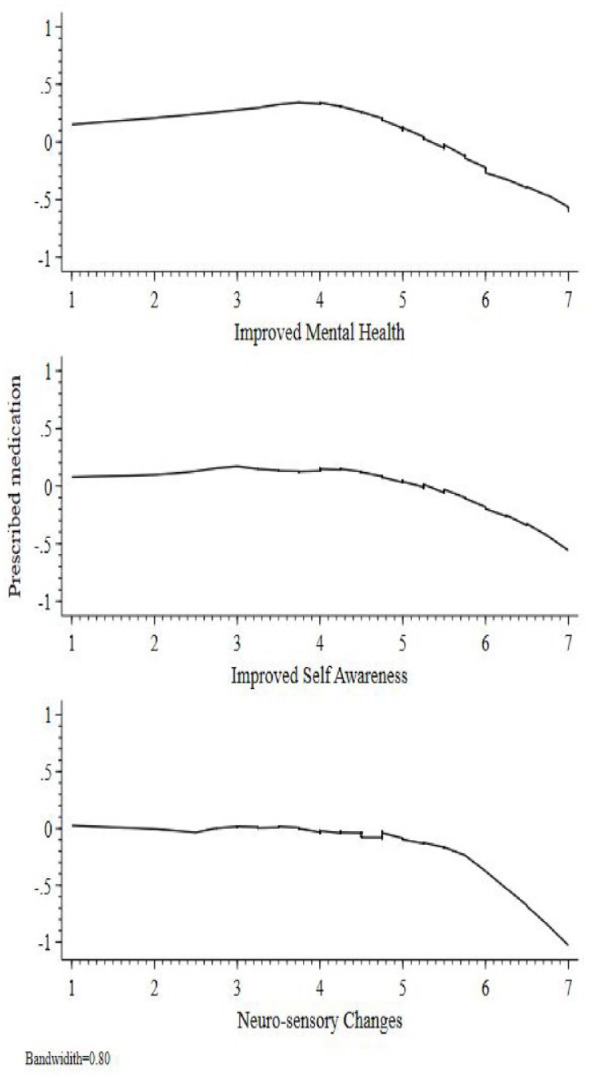
LOWESS smoother plots to model the association between current medication prescription and each of the new factors.

## Appendix A: GDS2020 item used for the exploratory factor analysis

Which of the following effects have you noticed as a result of your use of this psychedelic substance over the last 12 months on a scale of −3 to +3? (−3 strong negative consequences 0 no change +3 strongly positive n/a not applicable) For example, if you have a strong decrease in anxiety, you would put +3 for that question. If you had some positive changes in mood/reduced depression you may put in +2. As presented in the survey:

1. Change in overall symptoms of your psychiatric condition
2. Changes in mood or reduced depression
3. Changes in productivity, motivation or confidence
4. Changes in energy, alertness and/or focus
5. Changes in ability to control negative thoughts/persistent worryings
6. Changes in my tolerance towards others
7. Changes in feelings of frustration/anger
8. Changes in sight, smell or hearing
9. Changes in my understanding of why I feel the way I do
10. Changes in my understanding of my condition or how I relate to it
11. Changes in empathy, sociability and communication skills
12. Changes in concentration/ memory
13. Changes in anxiety, including social anxiety
14. Changes in sleep
15. Changes in my use of alcohol/other drugs
16. Changes in life priorities
17. Changes in self-identity
